# Assessing Rapid Adaptation Through Epigenetic Inheritance: A New Experimental Approach

**DOI:** 10.1111/pce.15220

**Published:** 2024-10-25

**Authors:** Meret Huber, Alexandra Chávez

**Affiliations:** ^1^ Institute of Organismic and Molecular Evolution Johannes Gutenberg University Mainz Mainz Germany

**Keywords:** epigenetic or nongenetic inheritance, epimutation and epialleles, experimental evolution, transgenerational plasticity

## Abstract

Epigenetic inheritance is hypothesized to lead to rapid adaptation, yet evidence is scarce, possibly because of the current experimental approaches. We propose a new approach to simultaneously assess whether species adapt through selection of epimutations or formation of stress‐induced epialleles.

## 1 Introduction

The hallmark of evolutionary biology posits that species adapt to stresses through selection of phenotypic variants that arise from DNA sequence variation. Yet, increasing evidence shows that DNA sequence variation is not the sole source of heritable phenotypic variation: for instance, flowers of natural toadflax mutants develop radial instead of bilateral symmetry because a floral symmetry gene became hypermethylated (Cubas, Vincent, and Coen 1999). Similarly, homeotic floral phenotypes in the oil palm arose during tissue culture because a homeotic gene is alternatively spliced upon spontaneous hypomethylation of an intron‐located transposon (Ong‐Abdullah et al. 2015). Furthermore, fruit ripening in a spontaneous tomato mutant is hampered because a gene of the SBP‐box family of transcription factors became hypermethylated and was thereby silenced (Manning et al. 2006). These examples highlight that mechanisms other than DNA sequence variation may lead to heritable, variable, and fitness‐relevant phenotypes, and as such they open up an exciting question: which role do mechanisms that generate heritable phenotypic variation in the absence of DNA sequence variation play in the adaptation to environmental stresses? Considering the ongoing loss of intraspecific genetic diversity and the rapid pace of environmental change (Des Roches et al. 2021; Sage 2020), answering this question is becoming increasingly relevant.

Heritable phenotypic variation that is not caused by DNA sequence changes may arise through so‐called ‘nongenetic’ or ‘epigenetic’ inheritance (Bonduriansky and Day 2009; O'Dea et al. 2016). Epigenetic inheritance often refers to mechanisms that alter gene expression across generations through genome‐associated mechanisms such as DNA methylation, histone modifications and small RNAs, whereas nongenetic inheritance includes any other mechanism such as the vertical transfer of microbes, nutrients or hormones (Bonduriansky and Day 2009). To improve readability, we here use the term ‘epigenetic’ to refer to both epigenetic and all other nongenetic mechanisms, regardless of how many generations the traits are inherited.

**Figure 1:**
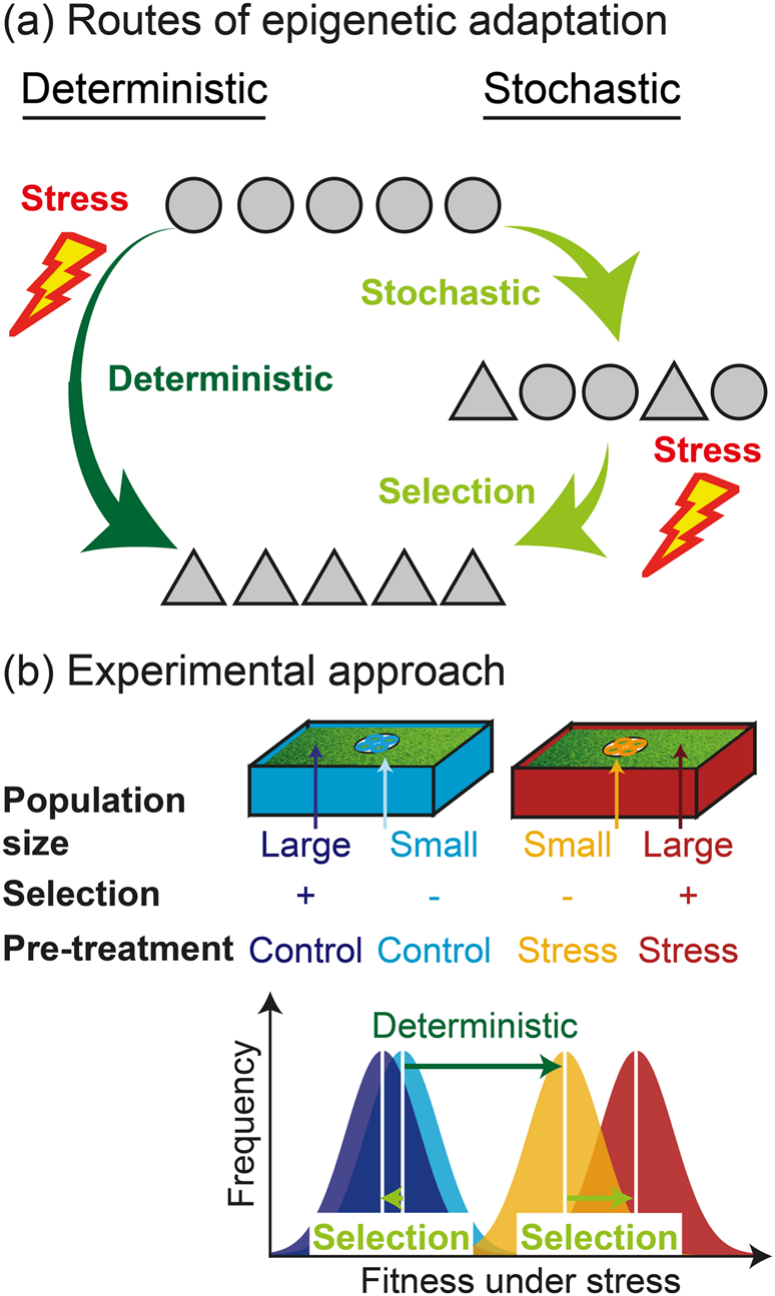
Experimental approach to tease apart the deterministic and stochastic route of adaptation through epigenetic inheritance. (a) Epigenetic inheritance may lead to rapid adaptation via deterministic epialleles (‘deterministic route’) or selection of stochastic epimutations (‘stochastic route’). The circles and triangles represent different individuals, with each shape depicting different epigenetic marks at the same locus. (b) To differentiate between the deterministic and stochastic routes, genetically uniform individuals are grown in different environments (‘pretreatments’) across multiple generations in both small and large populations. In small populations, drift will overcome the effect of selection, so only the deterministic route can occur. In large populations, selection can act, allowing both the deterministic and stochastic routes to occur. Consequently, the deterministic route can be detected by comparing small populations across different pretreatments, while the stochastic route can be discerned by comparing small and large populations within each pretreatment.

Many types of epigenetic marks share two features: first, epigenetic marks may change spontaneously over generations in a largely stochastic manner (Becker et al. 2011; Schmitz et al. 2011), and second, the environment may alter epigenetic marks, and thereby lead to predictable phenotypic variation (Wibowo et al. 2016; Zhang, Lang, and Zhu 2018). Consequently, epigenetic inheritance may lead to stress adaptation through two fundamentally different routes: the ‘stochastic’ and the ‘deterministic’ route (Figure 1a) (Baugh and Day 2020).

The stochastic route is comparable to the adaptive process that is based on DNA sequence variation. Here, spontaneous changes (‘epimutations’) arise either in the presence or absence of stress in a largely random manner. For instance, in *Arabidopsis thaliana*, variations in CG and to a much lower extent also CHG and CHH methylation (H = A, T, C) accumulate across generations (Becker et al. 2011; Denkena, Johannes, and Colomé‐Tatché 2021; Schmitz et al. 2011), and both the frequency and the spectrum of these spontaneous epimutations may be altered by stress (Jiang et al. 2014; Johannes and Schmitz 2019). In plants, these spontaneous epimutations are semistable, meaning that many epimutations are inherited across several generations but also frequently reverse (Johannes and Schmitz 2019). Although most of these epimutations likely do not affect phenotypes, a few epimutations, particularly those that cover differentially methylated regions rather than individual cytosines (Denkena, Johannes, and Colomé‐Tatché 2021), could increase the phenotypic diversity in genetically uniform individuals upon which natural selection can act.

The deterministic route, in contrast, is not comparable to the adaptive process that is based on DNA sequence variation. In the deterministic route, also described as transgenerational plasticity and reviewed in Anastasiadi et al. (2021), environmental stresses induce epigenetic variation and thereby lead to predictable epigenetic variations that are shared among individuals (‘deterministic epialleles’). For instance, in *A. thaliana*, environmental stresses remodel CHG and CHH methylation in specific repeat sequences, which is associated with altered expression of stress‐related genes nearby (Annacondia et al. 2021; Luna et al. 2011; Wibowo et al. 2016). Such stress‐induced epialleles are usually considered to have limited heritability, as they often vanish after maximally three offspring generations (Luna et al. 2011; Wibowo et al. 2016), and thus may be the consequence of the direct stress exposure during the early development of the offspring rather than being truly inherited (Grossniklaus et al. 2013). Recent evidence, however, indicates that deterministic epialleles may be substantially more stable than anticipated: in the duckweed *Lemna minor*, heat stress remodelled CHG methylation, and part of this variation was still observed after at least three clonal generations under control conditions (Van Antro et al. 2023). Similarly, in the closely related duckweed *S. polyrhiza*, copper excess induced variation in phenotypes and fitness that persisted for up to 10 clonal generations under control conditions (Huber, Gablenz, and Höfer 2021). Furthermore, in the sexually reproducing *A. thaliana*, abiotic stresses induced gene expression changes that were retained even for four generations (Lin et al. 2024). If deterministic epialleles are heritable across multiple generations, as these studies suggest, they may lead to rapid stress adaption even in the absence of selection.

## 2 The Problem: How to Tease Apart Adaptation From Stochastic and Deterministic Epigenetic Variation

While the stochastic route relies on random variation being selected upon, the deterministic route may allow populations to adapt in the absence of selection. Teasing apart these two routes is important in at least two reasons: first, the concept that species adapt through selection of beneficial variants is fundamental to evolutionary biology. The deterministic route of epigenetic adaptation would challenge this paradigm. Second, the deterministic route could be relevant for plant resistance in the field, particularly when intraspecific genetic variation is low, which is typical for crop species or endangered species. For instance, plants that are cultivated for seed production could be induced by a short‐term environmental stress, thereby altering offspring resistance (Vázquez‐Hernández et al. 2019). Similarly, endangered species could be exposed to mild levels of an environmental stress that the species likely experience in its habitat, which may increase offspring performance and the likelihood that the species persists. Thus, teasing apart the stochastic and deterministic routes is fundamental to evolutionary theory and could inform breeders and conservation biologists about whether epigenetic inheritance might improve crop performance and the resilience of endangered species. Yet, assessing and teasing apart the stochastic and deterministic route is experimentally challenging.

Experiments that aim to test the deterministic route are relatively common (Baugh and Day 2020; Huber, Gablenz, and Höfer 2021; Luna et al. 2011; Wibowo et al. 2016). Usually, genetically uniform organisms are grown for multiple generations under different environments (‘pretreatment’) as single descendants—thereby minimizing the confounding effects of genetic variation and selection of beneficial (epi‐) genetic variants (Baugh and Day 2020). Subsequently, individuals are grown for at least three generations under control conditions to ensure that the organisms were not directly exposed to stress during their early development (Heard and Martienssen 2014). Subsequently, fitness and epigenetic variation between the pretreatments are assessed and correlated to each other. Ideally, this experiment is accompanied with genetic or chemical manipulation of the epigenetic machinery and/or genes that are transgenerationally regulated to assess the underlying molecular mechanisms. While this approach is powerful to test the role of deterministic epialleles in stress adaptation, it does not allow to infer whether selection of stochastic variants may lead to stress adaptation.

Experiments that test whether stochastic epigenetic variants may mediate rapid stress adaption through natural selection are rather rare (Heckwolf et al. 2020; Huber, Gablenz, and Höfer 2021; Kronholm et al. 2017; Schmid et al. 2018), likely because of the experimental challenges. The most common approach is to grow large, genetically uniform populations for many generations under different environments (‘pretreatment’), subsequently grow individuals for a few generations under a shared control environment to erase environment‐induced effects, and then compare fitness and epigenetic variation between the populations of the different pretreatments. The limitation of this approach is that during the pretreatment, populations simultaneously experience both selection of stochastic variants as well as the induction of deterministic epialleles—and if the deterministic epialleles are heritable for multiple generations, and show unexpected temporal inheritance patterns (Bell and Hellmann 2019; Huber, Gablenz, and Höfer 2021)—variation between the populations could be either due to selection of stochastic variants or heritable deterministic epialleles. To assess whether stochastic epigenetic variation may mediate rapid adaption through natural selection, we thus need an approach in which selection of stochastic variants can take place while teasing apart the contribution of deterministic epialleles.

## 3 The Approach: Manipulating the Efficacy of Selection Through the Population Size

Here, we propose an experimental approach that unifies both above‐mentioned setups and thereby allows to simultaneously assess whether selection of stochastic variants and/or the formation of deterministic epialleles lead to rapid stress adaptation. One of the key differences between the stochastic and deterministic route is whether selection is needed—and as such, to differentiate among these two routes, one can manipulate the efficacy of selection. This can be achieved through the population size, as selection is most effective in large populations (McDonald 2019; Wahl, Gerrish, and Saika‐Voivod 2002). We therefore propose to subject populations for several generations to different environments (‘pretreatments’) at two different population sizes.

On the one hand, one should grow the species in population sizes sufficiently large that selection is effective. As space is limited, these populations will need to be grown either under recurring bottlenecks, or under constant population sizes by randomly removing individuals or—in case of annuals—establishing the same population size each generation (Schmid et al. 2018). It is difficult to predict how large the population size needs to be that selection is effective, because first, it is not trivial to infer the effective population size based on the—usually much larger—census population size (Frankham 2007), and second, it is largely unclear how quickly epimutations accumulate and to which extent epimutations improve fitness (Johannes and Schmitz 2019). To maximize the effective population size, one should keep the populations sizes as large as possible throughout the entire experiment. Particularly, the bottleneck size should not be smaller than 10% (Wahl, Gerrish, and Saika‐Voivod 2002). To increase the number of epimutations, one could use—if available—either epigenetic recombinant inbred lines (epiRILs) (Johannes et al. 2009), which harbour substantial epigenetic but not genetic variation, or genetic mutants in which a gene in the epigenetic machinery is either impaired or newly introduced, which should increase the number of newly emerging epigenetic variants. Given sufficient fitness‐relevant epigenetic variation and sufficiently large population size, these populations will undergo both the stochastic and deterministic route of adaptation.

On the other hand, one should grow the species in population sizes sufficiently small that selection is not effective, that is, that drift overcomes the effect of selection. This is achieved once the effective population size *N*_e_ < 1/s (s = selection coefficient) (McDonald 2019; Nielsen and Slatkin 2013); the smallest possible effective population size is obtained in single descendant lineages. Thus, the small population will evolve almost exclusively through drift, and thereby only undergo the deterministic route. Importantly, the large and small populations must be grown side by side in the very same environment to ensure that the stress level is equal for both population sizes and that the deterministic route is equally induced in the small and large populations.

After several generations of pretreatment, one can assess whether populations adapted to stress through the stochastic and/or deterministic route. To this end, individuals of the small and large populations of the different pretreatments are grown in a shared control environment for at least three generations to ensure that any variation is truly heritable. Subsequently, phenotypes and fitness under the different environments are compared. This allows differentiating whether selection of epigenetic variants and/or deterministic epialleles lead to rapid adaptation: if deterministic epialleles confer resistance, variation in resistance and epigenetic variation will arise between the small populations of the different pretreatments. If selection of epigenetic variants contributes to resistance, variation in resistance and in epigenetic variation will establish between the small and large population within each pretreatment (Figure 1b).

## 4 The Requirements: Is My Experimental System Suitable to Assess Adaptation to Stresses Through Epigenetic Inheritance Using the Proposed Approach?

While the proposed experiment is in principle applicable to species across the tree of life, the species under investigation should have following characteristics:

*Rapid reproduction and small body size*. As with all species used for experimental evolution, rapid reproduction and small body size are desirable.

*Low genetic variation*. Teasing apart genetic from epigenetic effects is notoriously difficult, see discussion below. We thus suggest minimizing genetic variation by using clonally reproducing organisms or organisms that are highly inbred; ideally, genetic mutation rates should be small to reduce genetic variation that arise during the experiment.

*Accurate assessments of fitness and epigenetic variation*. Measuring Darwinian fitness is critical when assessing adaptation—thus, direct fitness assessments (e.g., number of offspring) are preferred over indirect assessments such as phenotypes or altered performance of interacting species; such assessments are nevertheless valuable. Furthermore, the ability to measure epigenetic variation (e.g., DNA methylation, histone modification, small RNAs) in a largely unbiased, genome‐wide manner and to link these variations to specific genes, is desirable. Although these analyses often require a reference genome, new developments in high‐throughput sequencing will likely overcome some of the limitations of nonmodel species that do not have a high‐quality reference genome or whose genome is prohibitively large (Amarasinghe et al. 2020; Gawehns et al. 2022).

In principle, both asexually as well as sexually reproducing species can be used for the proposed approach. The mode of reproduction will, however, affect how likely either the deterministic or stochastic route is. The deterministic route appears to be more likely in asexually reproducing than sexually reproducing plants, because stress‐induced epigenetic mark seem to be more stable during mitotic, asexual reproduction compared to meiotic, sexual reproduction (Van Antro et al. 2023; Wibowo et al. 2016). The stochastic route is possible in both sexually as well as asexually reproducing plants, as in plants, spontaneous epimutations are heritable through both mitosis and meiosis (Becker et al. 2011; Yao, Schmitz, and Johannes 2021). Suitable asexually reproducing species include green algae, for example, *Chlamydomonas reinhardtii*, aquatic ferns such as *Azolla filiculoides*, *Salvinia cucullata*, *Marsilea quadrifolia* or *Regnellidium diphyllum*, and aquatic angiosperms including duckweeds or water pennyworts. Suitable sexually reproducing species include *A. thaliana, Lepidium sativum* and *Capsella bursa‐pastoris*.

## 5 Discussion

Here, we presented an experimental approach to test a long‐standing controversy: whether epigenetic inheritance can lead to rapid adaptation to environmental stresses through stochastic or deterministic epigenetic variation. One of the major differences between the stochastic and deterministic route is whether selection is needed for adaptation. Thus, to tease apart the stochastic and deterministic route, we suggest manipulating the efficacy of selection through the population size by growing both small and large populations for many generations in different environments and subsequently assess whether variation in phenotypes, fitness or epigenetic marks establish between environments or population sizes. The deterministic route would yield variations between the small populations of the different pretreatments, whereas the stochastic route would result in differences between the small and large population within each pretreatment.

The advantage of the approach is that we can accurately assess which variations between the large populations of the different environments are due to selection and which variations due to the induction of deterministic epialleles. Alternative approaches, in which the deterministic epialleles are inferred by growing small populations separated from the large ones do not accurately mimic the biotic and abiotic conditions of the large populations and thus likely do not induce deterministic epialleles to a similar extent in the small and large populations. Similarly, it is not sufficient to grow the small populations only for short term in the environment of the large populations, as the duration of the stress may alter the induction of epigenetic marks and phenotypes, as well as their heritability (Huber, Gablenz, and Höfer 2021). Thus, although growing the small populations alongside the large populations is tedious, this is needed to accurately assess whether variation in epigenetic marks or phenotypes is due to natural selection.

The disadvantage of the approach is that it relies on the assumption that the stochastic and deterministic route are separate, nonoverlapping entities. This, however, may not be the case. For instance, deterministic epialleles may be induced in a largely but not entirely deterministic manner: environmental stresses may simply increase the likelihood of an epiallele to appear but not cause all individuals changing their epigenetic status. Selection could thus still act on environment‐induced ‘deterministic’ epialleles. Similar accounts for the stochastic epimutations: environmental stresses may increase the likelihood that certain epimutations appear, associated with epigenetic hotspots (Hazarika et al. 2022; Zheng et al. 2017), and thus epimutations may not be entirely stochastic. Are the stochastic and deterministic route therefore two extremes of the same process? One argument holds against this: in *A. thaliana*, spontaneous epimutations mostly accumulate in the CG context (Becker et al. 2011; Denkena, Johannes, and Colomé‐Tatché 2021; Schmitz et al. 2011; van der Graaf et al. 2015) whereas environment‐induced epialleles are most prominent in the CHG and/or CHH context (Annacondia et al. 2021; Lin et al. 2022; Wibowo et al. 2016; Yadav et al. 2022; Zhou et al. 2019). This supports the idea that the stochastic and deterministic route are, at least in the context of DNA methylation in plants, two fundamentally different processes that can be teased apart using the proposed approach.

If the experiments reveals that the deterministic or stochastic route alters phenotypes or epigenetic marks, it will be critical to assess whether the underlying molecular mechanism is of genetic or epigenetic nature. Even when using highly inbred or clonally reproducing species, genetic mutations may accumulate and account for variation in phenotypes or epigenetic marks, particularly in the large populations and when using epigenetic mutants in which transposons are mobilized (Miura et al. 2001). We suggest the following experiments and analyses to tease apart genetic from epigenetic effects: first, unbiased methods (e.g., high‐throughput sequencing) should be deployed to screen for epigenetic variations. Second, transcriptome and proteome data should be generated to link fitness and phenotypes to gene expression and epigenetic variation. Third, differentially regulated genes or epigenetic machineries should be manipulated both genetically as well as chemically to test whether the identified genes or epigenetic machinery affect stress resistance or the process of stress adaptation. Ideally, site‐specific manipulation of DNA methylation of candidate genes is performed (Papikian et al. 2019). Fourth, phenotypes or fitness should be continuously assessed for multiple generations after stress release—if variations between groups have an epigenetic basis, the variation should diminish or at least change in some lineages over time. Fifth, high‐throughput sequencing should be deployed to screen for genetic variation in identified genomic regions. While in isolation none of these approaches will provide a clear answer whether genetic or epigenetic mechanisms are at play, combining these approaches will be powerful to disentangle genetic and epigenetic factors.

The above‐mentioned approach to identify and test the molecular mechanisms is promising particularly for the deterministic route, in which we expect all or at least most lineages carrying the same modifications. Identifying candidate genes or epigenetic marks involved in the stochastic route is more challenging, as we expect that each lineage carries different modifications. To narrow down the list of candidate genes that are epigenetically regulated, one could use a‐priory knowledge about which genes contribute to resistance or are at least which genes are induced by the stress. While this approach may overlook some genes that are epigenetically regulated, the approach is a reasonable first step to identify the most promising candidate loci.

If experiments suggest that epigenetic inheritance leads to rapid adaptation, the question arises how fast and effective such adaptation is compared to adaptation based on genetic variation—either standing genetic variation or de novo mutations. This question is important, because in nature, genetic variation is usually present. To answer this question, one could compare experimentally evolved populations in which standing genetic variation is initially either present or absent. Furthermore, one could reverse de novo mutations in experimentally evolved populations using genetic engineering to quantify the effect of the mutations on plant fitness. Such experiments are important to infer how important epigenetic inheritance is for rapid adaption in nature.

Taken together, we here provide an experimental framework to assess the relative contribution of deterministic epialleles and selection of stochastic epigenetic variants to rapid stress adaptation. Through this approach, carefully designed experiments can provide novel insights whether and how epigenetic inheritance may lead to rapid stress adaptation. Considering the on‐going loss of intraspecific genetic diversity and the rapid pace of environmental change, these insights may become increasingly relevant when assessing the species resilience during global change.

## Conflicts of Interest

The authors declare no conflicts of interest.

## Data Availability

The authors have nothing to report.
